# Supplementary material for: Association of gut microbiota with metabolism in juvenile Atlantic salmon
Source: Microbiome. 2020 Nov 16;8:160. doi: 10.1186/s40168-020-00938-2 (PMC7670802; doi:10.1186/s40168-020-00938-2)
Supplement: Supplementary file 2 — Additional file 1: Suppl. Table 1. Single-nucleotide polymorphisms (SNP) associated with the OTU variables. Suppl. Table 2. Regression estimates, standard errors, F- and p-values when regressing OTUs on growth, metabolism, and feed efficiency variables. The model also contained regression on day and random effects of animal (utilizing genomic relationships), and tank for which variance components are included. Suppl. Fig. 1. Genome-wide Manhattan plot for the different OTUs. The horizontal line represents the genome-wide Bonferroni -log10 (p) = 6.03 threshold. Suppl. Fig. 2. Q-Q plots from genome-wide association analyses of the different OTUs. [file 40168_2020_938_MOESM1_ESM.docx]

**SUPPLEMENTARY TABLES**

**Suppl. Table** **1.** Single-nucleotide polymorphisms (SNP) associated with the OTU variables.

| **Trait** | **Chr** | **SNP** | **bp** | **A1** | **A2** | **Freq** | **b** | **se** | ***p*** | **log10(*p*)** |
| --- | --- | --- | --- | --- | --- | --- | --- | --- | --- | --- |
| **OTU1** | 5 | ctg7180001925291_748_SCT | 27518103 | C | T | 0.10 | -0.51 | 0.12 | 2.49E-05 | -4.6 |
|  | 5 | ctg7180001311517_3981_SAG | 29290387 | G | A | 0.10 | -0.46 | 0.11 | 2.51E-05 | -4.6 |
|  | 5 | ctg7180001833723_2640_SAG | 56739234 | A | G | 0.01 | -1.44 | 0.28 | 4.53E-07 | -6.3 |
|  | 14 | ctg7180001903206_2590_SCT | 5592915 | C | T | 0.15 | -0.45 | 0.10 | 1.81E-06 | -5.7 |
|  | 19 | ctg7180001841090_969_SAG | 42066887 | G | A | 0.02 | -1.03 | 0.23 | 8.21E-06 | -5.1 |
|  | 19 | ctg7180001800309_4785_SCT | 67320228 | C | T | 0.09 | -0.55 | 0.13 | 1.70E-05 | -4.8 |
|  | 19 | ctg7180001645427_1176_SAG | 75579801 | A | G | 0.02 | -1.16 | 0.27 | 1.57E-05 | -4.8 |
| **OTU2** | 1 | ctg7180001908955_3685_SAG | 4697317 | G | A | 0.06 | -0.46 | 0.11 | 2.11E-05 | -4.7 |
|  | 5 | ctg7180001722113_3376_SGT | 25219965 | G | T | 0.01 | -1.05 | 0.21 | 7.26E-07 | -6.1 |
|  | 5 | ctg7180001672687_10612_SAG | 28647949 | A | G | 0.01 | -1.05 | 0.21 | 7.26E-07 | -6.1 |
|  | 6 | ctg7180001847439_3403_SAC | 32454164 | A | C | 0.03 | -0.59 | 0.14 | 2.32E-05 | -4.6 |
|  | 6 | ctg7180001828921_349_SAG | 64415855 | G | A | 0.01 | -0.96 | 0.20 | 1.30E-06 | -5.9 |
|  | 11 | ctg7180001900871_1769_SCT | 85379984 | T | C | 0.08 | -0.39 | 0.09 | 1.13E-05 | -4.9 |
|  | 12 | ctg7180001666651_1965_SAC | 12523402 | A | C | 0.03 | -0.70 | 0.16 | 1.56E-05 | -4.8 |
|  | 12 | ctg7180001840586_4279_SCT | 43076320 | C | T | 0.07 | -0.47 | 0.10 | 1.92E-06 | -5.7 |
|  | 12 | ctg7180001839373_5142_SAC | 54225053 | C | A | 0.01 | -1.14 | 0.21 | 8.12E-08 | -7.1 |
|  | 12 | ctg7180001484203_1236_SAC | 68394895 | C | A | 0.02 | -1.09 | 0.20 | 9.88E-08 | -7.0 |
|  | 15 | ctg7180001298386_4207_SGT | 20950022 | T | G | 0.02 | -0.95 | 0.21 | 3.77E-06 | -5.4 |
|  | 15 | ctg7180001878210_7094_SAG | 36237432 | A | G | 0.02 | -0.78 | 0.18 | 1.97E-05 | -4.7 |
|  | 22 | ctg7180001807745_17982_SAC | 34245759 | C | A | 0.02 | -1.08 | 0.21 | 1.40E-07 | -6.9 |
|  | 24 | ctg7180001301776_10058_SAG | 26185685 | A | G | 0.10 | -0.39 | 0.09 | 8.01E-06 | -5.1 |
|  | 29 | ctg7180001835668_5175_SAC | 35464325 | A | C | 0.04 | -0.76 | 0.13 | 6.34E-09 | -8.2 |
|  | 29 | ctg7180001540342_979_SAC | 37759078 | C | A | 0.13 | -0.32 | 0.07 | 1.51E-05 | -4.8 |
| **OTU5** | 7 | ctg7180001798708_11367_SCT | 14075405 | C | T | 0.51 | 0.27 | 0.06 | 2.56E-05 | -4.6 |
|  | 7 | ctg7180001798708_5931_SAG | 14080841 | G | A | 0.40 | -0.28 | 0.07 | 3.62E-05 | -4.4 |
|  | 15 | ctg7180001298386_4207_SGT | 20950022 | T | G | 0.02 | -1.15 | 0.26 | 1.41E-05 | -4.9 |
| **OTU6** | 3 | ctg7180001338507_1900_SCT | 12338431 | C | T | 0.36 | 0.17 | 0.04 | 1.47E-05 | -4.8 |
|  | 3 | ctg7180001916975_10480_SGT | 26091556 | T | G | 0.43 | 0.18 | 0.04 | 8.24E-06 | -5.1 |
| **OTU7** | 5 | ctg7180001841488_1545_SCT | 8907693 | C | T | 0.15 | 0.38 | 0.08 | 5.82E-06 | **-5.2** |

**Suppl. Table** **2.** Regression estimates, standard errors, *F*- and *p*-values when regressing OTUs on growth, metabolism, and feed efficiency variables. The model also contained regression on day and random effects of animal (utilizing genomic relationships), and tank for which variance components are included.

| **Dependent variable** | **Variables** | **Estimate** | **Stderr** | **F-value** | ***p*-value** | **Variance component** |
| --- | --- | --- | --- | --- | --- | --- |
| WG | Day | 3.338 | 0.177 | 355.73 | <0.005 |  |
|  | OTU1 | -0.324 | 0.376 | 0.15 | NS |  |
|  | OTU2 | 2.191 | 1.199 | 0.04 | NS |  |
|  | OTU3 | -1.352 | 0.624 | 4.66 | <0.05 |  |
|  | OTU5 | -0.743 | 0.535 | 1.78 | NS |  |
|  | OTU6 | -0.440 | 0.866 | 0.26 | NS |  |
|  | OTU7 | 0.179 | 0.492 | 0.30 | NS |  |
|  | Tank |  |  |  |  | 13.84 ± 3.25 |
|  | Animal |  |  |  |  | 5.54 ± 0.69 |
| RG | Day | 9.969 | 0.522 | 365.90 | <0.005 |  |
|  | OTU1 | 0.983 | 0.854 | 3.64 | NS |  |
|  | OTU2 | -3.500 | 2.715 | 0.01 | NS |  |
|  | OTU3 | 1.118 | 1.410 | 2.04 | NS |  |
|  | OTU5 | -0.149 | 1.214 | 0.06 | NS |  |
|  | OTU6 | 1.094 | 1.963 | 0.31 | NS |  |
|  | OTU7 | 2.121 | 1.117 | 3.29 | NS |  |
|  | Tank |  |  |  |  | 122.00 ± 26.75 |
|  | Animal |  |  |  |  | 14.56 ± 2.56 |
| AMC | Day | 0.270 | 0.027 | 101.92 | <0.005 |  |
|  | OTU1 | 0.004 | 0.006 | 0.01 | NS |  |
|  | OTU2 | -0.020 | 0.020 | 0.00 | NS |  |
|  | OTU3 | 0.006 | 0.010 | 0.42 | NS |  |
|  | OTU5 | 0.001 | 0.009 | 0.08 | NS |  |
|  | OTU6 | 0.010 | 0.014 | 0.49 | NS |  |
|  | OTU7 | 0.007 | 0.008 | 0.55 | NS |  |
|  | Tank |  |  |  |  | 0.48 ± 0.11 |
|  | Animal |  |  |  |  | 0.41x10^-3^ ± 1.03x10^-4^ |
| AMN | Day | 0.288 | 0.019 | 230.96 | <0.005 |  |
|  | OTU1 | 0.002 | 0.014 | 0.66 | NS |  |
|  | OTU2 | -0.048 | 0.045 | 0.59 | NS |  |
|  | OTU3 | 0.018 | 0.023 | 0.99 | NS |  |
|  | OTU5 | 0.015 | 0.020 | 0.85 | NS |  |
|  | OTU6 | 0.020 | 0.032 | 0.37 | NS |  |
|  | OTU7 | 0.017 | 0.018 | 0.57 | NS |  |
|  | Tank |  |  |  |  | 0.17 ± 0.04 |
|  | Animal |  |  |  |  | 0.41x10^-2^ ± 7.08x10^-4^ |
| ALC | Day | 0.088 | 0.028 | 9.78 | <0.005 |  |
|  | OTU1 | 0.004 | 0.004 | 0.29 | NS |  |
|  | OTU2 | -0.014 | 0.013 | 0.08 | NS |  |
|  | OTU3 | -0.004 | 0.007 | 0.34 | NS |  |
|  | OTU5 | 0.005 | 0.006 | 1.22 | NS |  |
|  | OTU6 | 0.009 | 0.010 | 0.89 | NS |  |
|  | OTU7 | 0.007 | 0.005 | 1.21 | NS |  |
|  | Tank |  |  |  |  | 1.83 ± 0.41 |
|  | Animal |  |  |  |  | 0.18x10^-3^ ± 4.52x^-4^ |

Suppl. Table 2. Continued

| **Dependent variable** | **Variables** | **Estimate** | **Stderr** | **F-value** | ***p*-value** | **Variance component** |
| --- | --- | --- | --- | --- | --- | --- |
| ALN | Day | 0.445 | 0.032 | 191.05 | <0.005 |  |
|  | OTU1 | 0.013 | 0.015 | 0.05 | NS |  |
|  | OTU2 | -0.061 | 0.047 | 0.00 | NS |  |
|  | OTU3 | 0.007 | 0.024 | 0.52 | NS |  |
|  | OTU5 | 0.012 | 0.021 | 0.88 | NS |  |
|  | OTU6 | 0.024 | 0.034 | 0.49 | NS |  |
|  | OTU7 | 0.034 | 0.019 | 2.58 | NS |  |
|  | Tank |  |  |  |  | 0.51 ± 0.11 |
|  | Animal |  |  |  |  | 0.37x10^-2^ ± 7.42x10^-4^ |
| AAC | Day | 0.082 | 0.019 | 17.95 | <0.005 |  |
|  | OTU1 | 0.007 | 0.003 | 1.39 | NS |  |
|  | OTU2 | -0.016 | 0.009 | 2.15 | NS |  |
|  | OTU3 | -0.001 | 0.005 | 1.31 | NS |  |
|  | OTU5 | 0.006 | 0.004 | 4.63 | <0.05 |  |
|  | OTU6 | -0.001 | 0.007 | 0.01 | NS |  |
|  | OTU7 | 0.010 | 0.004 | 8.53 | <0.005 |  |
|  | Tank |  |  |  |  | 0.90 ± 0.20 |
|  | Animal |  |  |  |  | 0.87x10^-4^ ± 2.21x10^-4^ |
| IFCR_AMC | Day | 0.231 | 0.020 | 130.26 | <0.005 |  |
|  | OTU1 | -0.024 | 0.023 | 2.34 | NS |  |
|  | OTU2 | 0.053 | 0.072 | 0.21 | NS |  |
|  | OTU3 | -0.020 | 0.037 | 1.32 | NS |  |
|  | OTU5 | 0.019 | 0.032 | 0.09 | NS |  |
|  | OTU6 | -0.009 | 0.052 | 0.03 | NS |  |
|  | OTU7 | -0.044 | 0.030 | 2.25 | NS |  |
|  | Tank |  |  |  |  | 0.19 ± 0.04 |
|  | Animal |  |  |  |  | 0.24x10^-2^ ± 1.08x10^-3^ |
| IFCR_AMN | Day | 0.554 | 0.047 | 138.65 | <0.005 |  |
|  | OTU1 | -0.086 | 0.049 | 1.71 | NS |  |
|  | OTU2 | 0.149 | 0.153 | 1.61 | NS |  |
|  | OTU3 | -0.049 | 0.079 | 1.18 | NS |  |
|  | OTU5 | 0.083 | 0.069 | 0.85 | NS |  |
|  | OTU6 | -0.057 | 0.111 | 0.26 | NS |  |
|  | OTU7 | -0.105 | 0.063 | 2.47 | NS |  |
|  | Tank |  |  |  |  | 1.01 ± 0.21 |
|  | Animal |  |  |  |  | 0.64x10^-2^ ± 4.43x10^-3^ |
| IFER_AMC | Day | 0.365 | 0.021 | 297.49 | <0.005 |  |
|  | OTU1 | 0.043 | 0.026 | 5.55 | <0.03 |  |
|  | OTU2 | -0.101 | 0.083 | 0.41 | NS |  |
|  | OTU3 | 0.018 | 0.043 | 1.34 | NS |  |
|  | OTU5 | -0.023 | 0.037 | 0.03 | NS |  |
|  | OTU6 | 0.035 | 0.060 | 0.34 | NS |  |
|  | OTU7 | 0.081 | 0.034 | 5.22 | <0.03 |  |
|  | Tank |  |  |  |  | 0.20 ± 0.04 |
|  | Animal |  |  |  |  | 0.43x10^-2^ ± 1.51x10^-3^ |
| IFER_AMN | Day | 0.149 | 0.009 | 273.21 | <0.005 |  |
|  | OTU1 | 0.020 | 0.009 | 3.89 | <0.05 |  |
|  | OTU2 | -0.026 | 0.027 | 2.86 | NS |  |
|  | OTU3 | 0.001 | 0.014 | 0.64 | NS |  |
|  | OTU5 | -0.016 | 0.012 | 0.79 | NS |  |
|  | OTU6 | 0.008 | 0.020 | 0.16 | NS |  |
|  | OTU7 | 0.027 | 0.011 | 5.83 | <0.03 |  |
|  | Tank |  |  |  |  | 0.04 ± 7.86x10^-3^ |
|  | Animal |  |  |  |  | 0.29x10^-3^ ± 1.51x10^-4^ |

SUPPLEMENTARY FIGURES


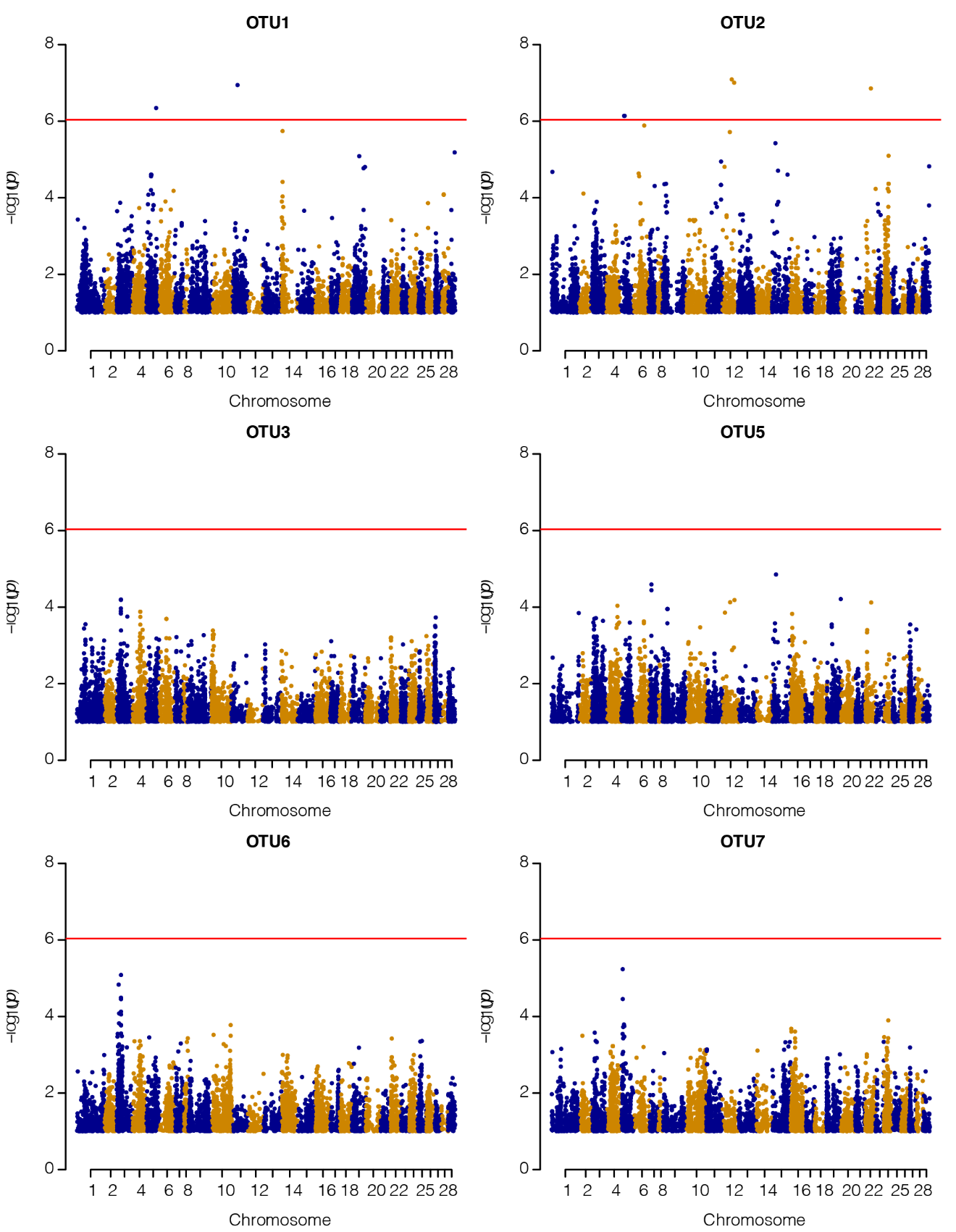


**Suppl. Fig.** **1. Genome-wide Manhattan plot for the different OTUs**. The horizontal line represents the genome-wide Bonferroni -log_10_ (*p*) = 6.03 threshold.


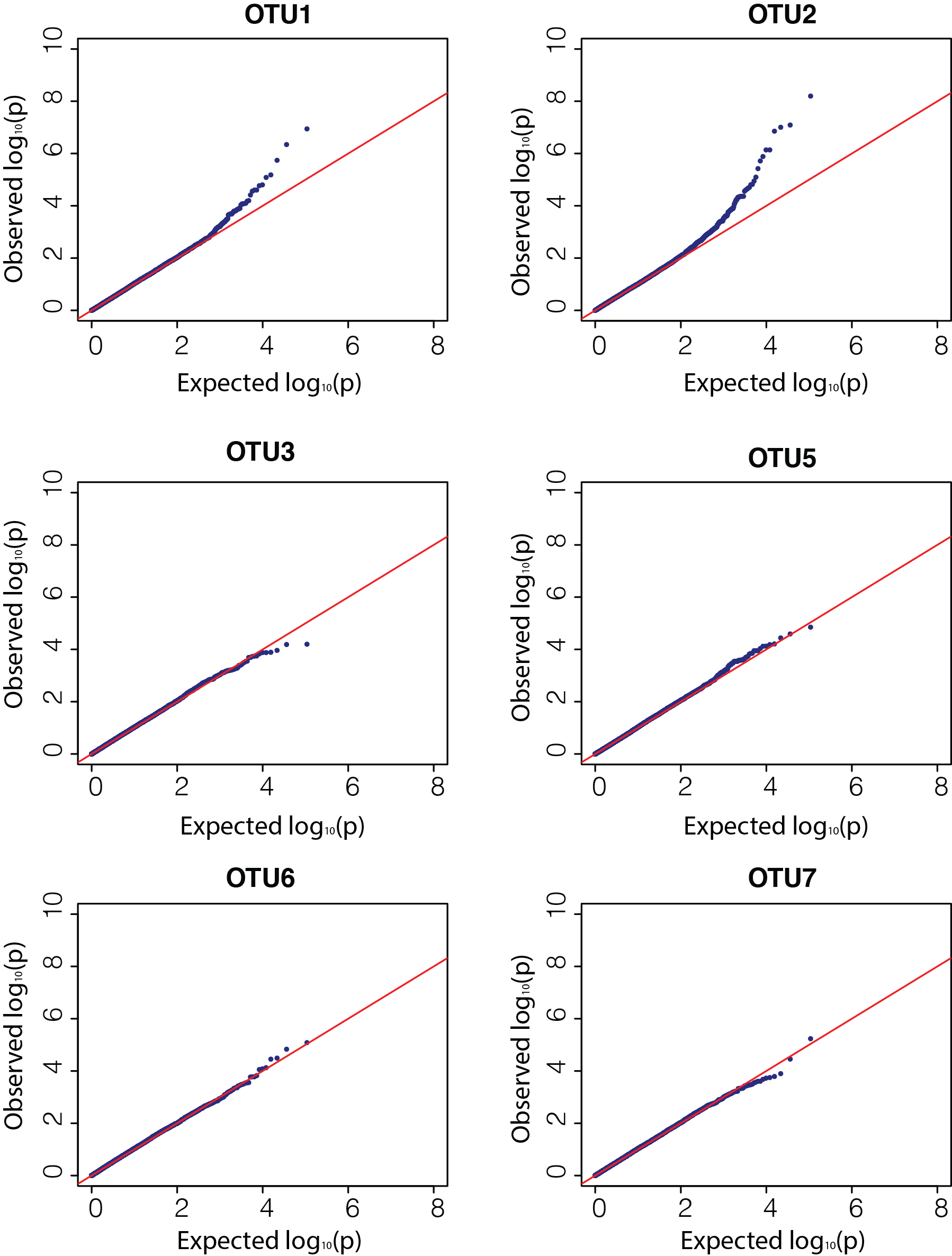


Suppl. Fig. 2. Q-Q plots from genome-wide association analyses of the different OTUs.
